# Bridging the gap: modern pharyngolaryngoesophagectomy techniques and the rise of neoadjuvant/immunotherapy approaches

**DOI:** 10.1186/s12957-026-04519-9

**Published:** 2026-07-31

**Authors:** Freideriki Nteka, Tania Triantafyllou, Ioannis Rouvelas, Ioannis Gkoutziotis, Panagiotis Sakarellos, Maria Tolia, Ioannis Karavokyros, Konstantinos Mpallas, Dimitrios Schizas

**Affiliations:** 1 First Department of Surgery, National and Kapodistrian University of Athens, Laikon General Hospital, Athens, Greece; 2 First Propaedeutic Department of Surgery, National and Kapodistrian University of Athens, Hippocration General Hospital, Athens, Greece; 3 Department of Upper Abdominal Surgery, Center for Digestive Diseases, Division of Surgery and Oncology, Department of Clinical Science, Intervention and Technology (CLINTEC), Karolinska University Hospital, Karolinska Institutet, Huddinge, Stockholm, 141 86 Sweden; 4 Fifth Department of Surgery, Aristotle University of Thessaloniki, Hippocration Hospital, Thessaloniki, Greece; 5 Department of Radiotherapy, School of Medicine, University of Crete, Heraklion, Greece

**Keywords:** Pharyngolaryngoesophagectomy, Neoadjuvant therapy, Immunotherapy, Morbidity, Mortality

## Abstract

**Background:**

The purpose of this review is to outline the evolution and changing role of pharyngolaryngoesophagectomy (PLE) in the organ-preservation era, and to review advances, including minimally invasive techniques, neoadjuvant therapy, and immunotherapy, and their impact on outcomes in advanced laryngeal, hypopharyngeal, and cervical esophageal cancers.

**Main body:**

A narrative review of the literature from 2000 to the present was performed, focusing on studies of surgical innovations, oncologic outcomes, quality of life, and combined treatment strategies in PLE. Historically, PLE was associated with high morbidity and mortality. However, advances in surgical techniques, reconstructive methods, and perioperative care have significantly improved its safety and functional outcomes. Minimally invasive and hybrid PLE approaches reduce pulmonary complications and speed recovery without compromising oncologic outcomes. Chemoradiation has become the first-line treatment for many advanced cases, with PLE now reserved for selected cervical esophageal tumors or persistent disease after radiation. Neoadjuvant chemotherapy or chemoradiation can shrink tumors, improve operability, and sometimes allow organ preservation. Immunotherapy before surgery improves pathological response rates and disease-free survival. Recent series report 5-year survival of 20–40% after PLE, and most patients regain swallowing and voice function with rehabilitation.

**Conclusion:**

In the organ-preservation era, PLE remains important for certain advanced or treatment-resistant cases. Its role is defined by a multidisciplinary, personalized approach and limited to cases where surgery is essential, often combined with systemic therapy. Ongoing advances in patient selection, minimally invasive techniques, and immunotherapy are expected to further refine PLE’s role and improve outcomes.

**Supplementary Information:**

The online version contains supplementary material available at 10.1186/s12957-026-04519-9.

## Background

This narrative review examines the contemporary role of pharyngolaryngoesophagectomy in the management of advanced squamous cell carcinoma of the larynx, hypopharynx, and cervical esophagus. In the organ-preservation era, its role has become increasingly selective and is now more clearly defined within multidisciplinary treatment algorithms, while advances in reconstruction, perioperative care, and minimally invasive surgery have improved its safety and functional outcomes. In parallel, neoadjuvant therapy and immunotherapy are reshaping treatment strategies and may further refine the role of surgery in this setting.

## Main text

### Introduction

Squamous cell carcinoma (SCC) is the predominant histologic subtype of malignancies affecting the hypopharynx, larynx, and cervical esophagus. These tumors share common etiologic factors, most notably tobacco and alcohol exposure, and are often characterized by aggressive local invasion, submucosal spread, and a high propensity for regional nodal metastasis [1]. Advanced-stage SCC in these sites frequently extends across anatomical boundaries, necessitating en bloc resection for oncologic clearance. Despite advances in organ-preservation protocols, SCC involving the pharyngolaryngeal-esophageal axis continues to present a therapeutic challenge, and pharyngolaryngoesophagectomy (PLE) remains an important consideration in selected cases where non-surgical approaches are unlikely to achieve durable disease control. In particular, esophageal squamous cell carcinoma (ESCC) of the cervical esophagus represents a distinct clinical entity, comprising a small minority of all esophageal cancers but carrying a poor prognosis. Due to its anatomic proximity to the larynx and hypopharynx, curative resection almost invariably requires PLE, and while definitive chemoradiotherapy is often employed with organ-preservation intent, surgical resection continues to provide an essential option for selected patients with localized or treatment-refractory disease [2]. PLE is an extensive surgical procedure involving en bloc resection of the pharynx, larynx, and part or all of the esophagus. It is typically reserved for selected advanced upper aerodigestive tract cancers that extend across these anatomical regions. Historically, PLE has been regarded as a procedure of last resort because of both its functional consequences, including loss of natural voice and profound alteration of swallowing, and its substantial perioperative risk. This perception was not based solely on the functional consequences of laryngeal and esophageal removal, but also on the morbidity inherent to a combined head and neck, thoracic, and reconstructive operation. Reported complications include pulmonary infection or respiratory failure, cardiopulmonary failure, arrhythmias, myocardial infarction or heart failure, renal dysfunction or acute kidney injury, anastomotic leakage, conduit necrosis, wound complications, and sepsis. These risks are particularly relevant in patients with advanced aerodigestive tract malignancy, who frequently present with malnutrition, tobacco- and alcohol-related comorbidity, prior radiotherapy or chemoradiotherapy, and limited cardiopulmonary reserve [3]. Consequently, the historical morbidity of PLE has strongly influenced multidisciplinary efforts to develop organ-preservation strategies and to reserve surgery for carefully selected patients in whom non-surgical treatment is unlikely to provide durable oncologic control or acceptable function [4]. In the modern era of organ preservation, wherein many laryngeal and hypopharyngeal cancers are treated with definitive chemoradiation to avoid total laryngectomy, the role of PLE has evolved. Contemporary indications are more narrowly defined, often centering on tumors where organ-preserving therapies are unlikely to achieve cure or acceptable post-treatment function, including selected primary tumors with extensive cervical esophageal involvement and recurrent or persistent disease after radiotherapy or chemoradiotherapy. Over recent decades, advances in surgical technique, reconstruction, perioperative care, nutrition, and adjuvant therapy have improved the safety and functional potential of PLE [5]. Modern reconstructive strategies may restore alimentary continuity, and many patients can ultimately resume oral intake; however, swallowing recovery remains variable and may be limited by tumor extent, prior irradiation, reconstruction type, anastomotic complications, strictures, reflux, aspiration risk, or prolonged tube dependence [6, 7]. Voice rehabilitation using an electrolarynx, esophageal or gastric speech, or tracheoesophageal prosthetic rehabilitation is feasible in selected patients, but communication outcomes after extended pharyngolaryngeal resection remain heterogeneous and should therefore be regarded as an important rehabilitative goal rather than a predictable functional endpoint [8, 9].

Herein, this narrative review provides a comprehensive overview of PLE for SCC involving the larynx, hypopharynx, and cervical esophagus, with emphasis on the contemporary literature from 2000 onward. It synthesizes the historical development of PLE, delineates current indications in both primary and salvage settings, and expands on modern operative and reconstructive refinements, including advances in perioperative care, microvascular and visceral conduit reconstruction, minimally invasive and hybrid approaches, highlighting postoperative outcomes, complication profiles, functional rehabilitation, long-term survival, and patient-centered quality of life. Finally, the growing role of multimodal management, with emphasis on neoadjuvant systemic therapy and the emerging integration of immunotherapy, in optimizing patient selection, improving functional outcomes, and refining the role of PLE, is discussed.

### Historical background

The concept of en bloc resection of the larynx, pharynx, and esophagus for cancer has its roots in the late 19th and mid-20th centuries. Early attempts at pharyngoesophageal resection were fraught with technical challenges and prohibitive mortality. It was not until the mid-20th century that PLE became a viable even though high-risk option, mostly due to improvements in anesthesia, surgical techniques, and perioperative care [10]. Total PLE as a formal procedure was introduced around 1960, initially accompanied by very high perioperative mortality rates exceeding 30%. In these early decades, two-stage reconstructions such as the Wookey two-step skin flap technique and other regional flaps were employed for pharyngoesophageal defects. By the 1960–1990 s, visceral transposition techniques became standard: gastric pull-up (pharyngogastric anastomosis) and colon interposition were favored to replace the resected esophagus and pharynx. Concurrently, pedicled regional flaps (e.g. deltopectoral and pectoralis major flaps) were used to assist in closure of large neck wounds or reinforce repairs. The late 1980s marked a revolution in reconstruction with the advent of microvascular free tissue transfer. Fasciocutaneous free flaps such as the radial forearm free flap and the anterolateral thigh (ALT) flap began to be used for pharyngoesophageal reconstruction, offering well-vascularized tissue to reconstruct partial pharyngeal or short-segment circumferential esophageal defects. Free jejunal interposition grafts also emerged as a figurative option for esophageal replacement after PLE, first described by Seidenberg in 1959 and increasingly utilized due to beneficial outcomes compared to earlier methods. These advancements in reconstructive strategies provided a tailored approach that dramatically decreased the morbidity of reconstruction, while they enhanced the success rates of restoring alimentary continuity and airway safety [11]. Although modern reconstruction, perioperative care, and structured rehabilitation have improved functional recovery after PLE, swallowing and communication outcomes remain variable. Contemporary series suggest that many patients ultimately regain oral alimentation, with complete oral intake reported in approximately 82.7% after pharyngolaryngoesophagectomy with gastric pull-up reconstruction, although recovery may be limited by anastomotic complications, strictures, prior irradiation, and the extent of resection. Voice rehabilitation is feasible through tracheoesophageal puncture (TEP), tracheogastric puncture, or electrolarynx use, but functional communication is not uniformly achieved. Accordingly, voice restoration should be presented as an important rehabilitative objective rather than a consistently predictable outcome [6, 8]. By the 21st century, mortality and complication rates for PLE had noticeably improved. Reports from the 1990s noted that perioperative mortality had fallen below 5–10% in high-volume centers, and anastomotic leak rates, once as high as 20–30%, had decreased to under 10%. The procedure that was once deemed almost prohibitive began to be performed with acceptable safety [12]. The historical evolution of PLE is also intertwined with changes in nonsurgical therapy. The 1990s and 2000s organ-preservation protocols (concurrent chemoradiation and induction chemotherapy strategies) became standard for many advanced laryngeal/hypopharyngeal cancers, thereby reducing the frequency of upfront PLE. The role of PLE shifted more towards to salvage surgery and thus PLE transformed from a high-risk, ultimate operation to a more refined procedure supported by improved reconstruction alternatives as well as perioperative care. This historical evolution informs contemporary practice, in which PLE is reserved for carefully selected patients within a multidisciplinary framework [13].

### Technical steps and evolving strategies in PLE

In the traditional open approach, PLE is performed through combined cervical and abdominal incisions, with or without a thoracic incision, to access all involved anatomical regions. The operation entails en bloc removal of the larynx with partial pharyngectomy at the cervical level, combined with complete esophagectomy. Mobilization of the cervical and upper thoracic esophagus is performed through the cervical incision, while the distal esophagus and stomach are dissected through an abdominal approach. Following resection, digestive continuity is typically restored by constructing a cervical anastomosis. Given the magnitude of this surgery, open PLE has traditionally carried significant morbidity and poses complex reconstructive challenges. Immediate reconstruction is most commonly achieved using a gastric conduit, or gastric pull-up, owing to its dependable vascularity and adequate length. In select cases, alternative conduits such as colon interposition or a free jejunal graft may be employed, often with microvascular augmentation to enhance perfusion and reduce the risk of graft necrosis or anastomotic leak [14]. Perioperative morbidity remains a defining concern in PLE and should be considered alongside the technical aspects of resection and reconstruction. In addition to cervical anastomotic leak, conduit necrosis, wound infection, chylothorax, and sepsis, cardiopulmonary complications represent a major source of early postoperative morbidity and mortality. Pulmonary complications may include atelectasis, pneumonia, aspiration, pleural effusion, prolonged ventilatory support, and respiratory failure, whereas cardiovascular events may include arrhythmias, myocardial ischemia, heart failure, hemodynamic instability, and thromboembolic events. These risks are amplified by malnutrition, tobacco-related pulmonary disease, alcohol-related comorbidity, prior radiotherapy or chemoradiotherapy, prolonged operative time, and the physiologic burden of combined cervical, thoracic, and abdominal surgery. Reported mortality varies across series according to case mix, reconstruction type, institutional volume, and whether surgery is performed in the primary or salvage setting; therefore, perioperative mortality should be interpreted in the context of patient selection and center experience [15]. These considerations support the need for careful preoperative cardiopulmonary assessment, nutritional optimization, multidisciplinary planning, meticulous conduit handling, early recognition of anastomotic or conduit-related complications, and standardized postoperative respiratory care.

Over the past two decades, PLE has evolved through refinements in both access and reconstruction, through minimally invasive approaches aimed at reducing surgical trauma, pulmonary morbidity, postoperative pain, and length of hospitalization. Building on traditional open cervical resection combined with thoracoscopic and laparoscopic techniques in esophageal surgery, minimally invasive PLE (MIPLE) has emerged as a feasible alternative [16]. Early reports demonstrated the technical feasibility of combining thoracoscopic esophagectomy and laparoscopic gastric mobilization with standard cervical resection, and comparative data from total PLE (TPLE) and broader esophagectomy literature suggest that minimally invasive approaches may reduce blood loss, wound morbidity, pulmonary complications, and hospital stay in selected cohorts [17]. Nevertheless, MIPLE does not eliminate the inherent complexity of the operation. Morbidity remains largely determined by the cervical phase, as pharyngogastric leakage, tracheal stump complications, salivary contamination, and conduit perfusion deficits may occur despite minimally invasive thoracic or abdominal access [18]. While robot-assisted esophagectomy may offer technical advantages, including enhanced visualization, improved dexterity, and more precise mediastinal dissection and lymphadenectomy, data directly evaluating robotic PLE remain scarce, warranting caution when extrapolating from the broader robotic esophagectomy literature. Available data suggest that robotic approaches may reduce some pulmonary or cardiopulmonary complications compared with open esophagectomy in selected settings, but they have not consistently demonstrated reductions in all major complications or perioperative mortality when compared with conventional minimally invasive esophagectomy [19]. Thus, MIPLE and robotic approaches should be viewed as evolving, morbidity-modifying strategies rather than definitive solutions to the perioperative risk of PLE, and their use is best reserved for high-volume centers with integrated head and neck, thoracic, abdominal, microvascular, anesthetic, and critical-care expertise. Currently, MIPLE is generally reserved for selected tumors requiring combined resection of the larynx, pharynx, and cervical or upper thoracic esophagus, predominantly in high-volume centers with expertise in both head and neck and esophageal surgery [20]. In other words, MIPLE represents an evolution rather than a replacement of the traditional open technique, contributing to improved perioperative outcomes while preserving oncologic principles in the multidisciplinary management of advanced hypopharyngeal and cervical esophageal cancers [21].

### Indications for PLE in the organ preservation era

In the era of organ preservation, most locally advanced laryngeal and hypopharyngeal squamous cell carcinomas are initially treated with definitive radiotherapy, concurrent chemoradiotherapy, or induction chemotherapy followed by response-adapted radiotherapy or chemoradiotherapy, with the aim of avoiding total laryngectomy while maintaining oncologic control. The success of these strategies, however, is highly dependent on tumor site, T stage, cartilage invasion, baseline laryngopharyngeal function, nodal burden, and treatment-related toxicity [22]. In selected hypopharyngeal cohorts, induction chemotherapy-based organ-preservation protocols have achieved laryngeal preservation without clear compromise of survival compared with immediate surgery; however, long-term outcomes remain guarded [23].

PLE remains indicated for selected advanced laryngeal and hypopharyngeal cancers with extension to the post-cricoid region, esophageal inlet, or cervical esophagus, particularly when durable control with organ-preserving therapy is unlikely. In these cases, submucosal upper esophageal spread or post-cricoid involvement may preclude adequate clearance by conventional laryngectomy or partial pharyngectomy alone [24]. For selected T4 tumors with cervical esophageal extension, cartilage destruction, or poor baseline function, surgery-based management may provide the most dependable means of achieving negative margins and sustained locoregional control [25]. In this setting, total laryngopharyngectomy combined with esophagectomy provides en bloc clearance of disease extending beyond the limits of standard laryngectomy or partial pharyngectomy [26]. Moreover, cervical esophageal squamous cell carcinoma represents a distinct indication for PLE because of its proximity to the hypopharynx, larynx, trachea, and recurrent laryngeal nerves. PLE may also be indicated for synchronous head and neck and esophageal SCC, as it enables single-stage en bloc resection of both malignancies and may provide favorable outcomes in carefully selected patients, despite substantial morbidity [27]. Salvage surgery represents another key indication, particularly for recurrent or persistent disease after primary chemoradiotherapy involving the cervical esophagus or diffusely affecting the pharyngolaryngeal region, where total laryngectomy alone would be oncologically insufficient. Although organ-preservation protocols are widely used in laryngeal and hypopharyngeal cancers, persistent or recurrent disease remains challenging, and prior radiotherapy may complicate subsequent surgery through fibrosis, impaired vascularity, and delayed wound healing. In this context, salvage PLE could provide long-term disease control in selected patients through en bloc removal of the irradiated larynx, pharynx, and esophagus, although it carries increased complication risk and often requires vascularized flap reconstruction to support healing and reduce fistula or wound breakdown [28].

Although definitive chemoradiotherapy is generally favored as first-line treatment due to its organ-preserving potential and survival outcomes comparable to surgery in selected patients, results remain heterogeneous and stage dependent. Contemporary series report 5-year overall survival after definitive chemoradiotherapy ranging from approximately 28.3% to 59.4%, with lower complete response rates in T4 disease than in T1–3 tumors. Consequently, surgery remains appropriate when chemoradiotherapy is contraindicated, unsuccessful, or unlikely to provide durable control, particularly in bulky, deeply invasive, symptomatic, or salvage settings [29]. On the other hand, immunotherapy has expanded the multimodal treatment landscape for head and neck SCC. However, immune checkpoint inhibition should not currently be considered a definitive organ-preservation substitute for PLE in extensive pharyngolaryngeal or cervical esophageal involvement. Its role remains complementary, while current evidence does not establish immunotherapy as a reliable replacement for en bloc pharyngolaryngoesophageal resection when surgery is required for oncologic clearance [30].

Non-operative strategies should also be interpreted by their risks of treatment-related mortality and failure. Although organ-preservation protocols may achieve meaningful laryngeal preservation and survival in selected patients, relapse and salvage surgery remain clinically relevant. Population-based esophageal cancer data report early mortality after definitive chemoradiotherapy of approximately 2.5% at 30 days and 8.3% at 90 days, while complete response is markedly less frequent in T4 than in T1–3 cervical esophageal SCC. These considerations emphasize the need for careful patient selection and preserve the role of PLE in cases where non-operative therapy is unlikely to achieve sustained disease control [31].

The decision to undertake PLE should therefore be individualized through multidisciplinary deliberation, integrating tumor extent, expected response to non-operative treatment, baseline swallowing and airway function, comorbidity, nutritional status, prior treatment, patient preference, and institutional expertise, thus balancing the potential benefits of non-surgical modalities against the risks inherent to extensive surgery. PLE is generally reserved for: (1) tumors with cervical esophageal extension or other anatomical features associated with poor chemoradiotherapy response; (2) bulky or infiltrative cervical esophageal SCC in which definitive chemoradiotherapy is contraindicated, has failed, or is unlikely to achieve durable control; (3) synchronous head and neck and esophageal SCC amenable to single-stage resection; and (4) salvage of persistent or locally recurrent disease after failed organ-preservation therapy. As organ-preserving treatments improve, the indications for primary PLE have narrowed, but PLE remains indispensable for certain advanced cases where it offers the best curative course [24, 30].

### Postoperative survival and quality of life

Nowadays, PLE operative mortality has declined to acceptable levels, and a meaningful proportion of patients has achieved prolonged survival. For example, a series of 208 patients undergoing PLE with gastric pull-up reconstruction reported no intraoperative deaths and only four in-hospital fatalities (∼2%). Likewise, reports of MIPLE have documented zero 30-day mortality [32]. Although operative mortality after PLE is now generally in the low-single-digit range, morbidity remains high. Complication rates typically range from 40% to 69% and the risk of leaks or fistulas (5–10%), and respiratory issues after PLE are emphasized, while preoperative radiotherapy and poor patient condition further increase complication rates [33]. Such events predispose to pharyngocutaneous or tracheoesophageal fistulation and commonly delay the resumption of oral feeding. Anastomotic stricture is reported in about 11% of patients, most often after postoperative radiotherapy, and typically necessitates repeated endoscopic dilations. Conduit and wound complications vary by technique, cervical skin-flap necrosis was the most frequent event (up to 34%), following wound infection in 4% with occasional reoperation. Respiratory morbidity remains prominent, with pneumonia or respiratory failure in approximately 10–15% of patients [34].

Long-term survival following PLE remains limited, largely reflecting the advanced stage and aggressive biology of the tumors that necessitate this procedure. Most contemporary series report 5-year overall survival (OS) in the range of 20–40%, with median survival typically between 17 and 30 months. Survival outcomes after PLE are broadly comparable to those achieved with definitive chemoradiotherapy for cervical esophageal and advanced hypopharyngeal cancers (5-year OS ~ 20–35%), yet non-surgical approaches are often favored for their quality-of-life benefits, while surgery remains justified in selected patients likely to achieve durable control [35].

Despite these challenges, many long-term survivors report acceptable functional status. Overall quality of life is generally favorable, with patients typically reporting better outcomes in domains related to swallowing and nutritional independence. Post-gastrectomy sequelae such as esophageal reflux and dumping syndrome may occur after gastric pull-up; however, most patients adapt with dietary modifications [36]. Notably, communication is restored through alternative methods like esophageal speech, electrolarynx, or tracheoesophageal puncture [37].

Quality of life is crucially contingent upon the avoidance of major complications. Anastomotic leaks typically necessitate weeks of non-oral (enteral) feeding, intensive wound care, and occasionally reoperation, resulting in nutritional compromise and delayed initiation of speech and swallowing therapy [38]. Adjuvant radiotherapy—commonly required in advanced disease—further elevates stricture risk and can induce chronic edema and fibrosis, thereby exacerbating dysphagia. Consequently, optimal recovery depends on coordinated multidisciplinary management, including early speech–language pathology involvement, structured swallowing exercises, individualized nutritional support, and comprehensive psychosocial care [39].

Among carefully selected patients, PLE can achieve acceptable long-term function, with most survivors regaining adequate swallowing and nutritional autonomy without permanent enteral support, although communication and social functioning often remain limited. Given that 5-year survival is broadly similar with surgery and definitive chemoradiotherapy (~ 20–35%), treatment selection should balance expected oncologic control against procedure-related morbidity and functional quality-of-life outcomes [40]. Multidisciplinary support, including speech and swallowing therapy, nutritional guidance and psychosocial services, is essential, and in carefully selected patients, upfront surgery can yield acceptable functional results while avoiding the added morbidity of salvage procedures in irradiated fields [41].

### Multimodal treatment strategies and the role of neoadjuvant therapy

Treatment of cancers that may require PLE involves a comprehensive multimodal strategy, combining surgical resection with radiotherapy, chemotherapy, and emerging targeted systemic approaches. The optimal therapeutic approach is determined on an individual basis, guided by tumor site, stage, biological behavior, and patient-related variables [3]. In recent years, neoadjuvant therapy has been introduced as part of this multimodal approach, bringing significant benefits in terms of both survival and functional preservation. Neoadjuvant treatment can consist of chemotherapy alone (induction chemotherapy) or chemoradiotherapy (CRT). Organ-preservation protocols, referring to induction chemotherapy–guided radiotherapy or concurrent CRT, can achieve survival comparable to upfront PLE in advanced laryngeal/hypopharyngeal cancer, even when cervical esophageal extension is present [42]. A response-adapted strategy using induction chemotherapy identifies candidates for definitive CRT, yielding substantial laryngeal and esophageal preservation without increasing severe complications, while triaging non-responders to surgery. Accordingly, induction chemotherapy is employed as a practical selection tool to individualize treatment and maximize functional preservation if oncologically feasible [43]. Beyond its role in organ-preservation decision-making, induction chemotherapy may facilitate tumor downstaging before surgery, potentially converting borderline unresectable disease to resectable disease, simplifying subsequent resection, and addressing micrometastatic disease [44].

As evidenced by randomized trials and meta-analyses, both neoadjuvant induction chemoradiation and chemotherapy have proven efficacy in squamous cell carcinoma. More specifically, pioneering trials in laryngeal and hypopharyngeal cancers demonstrated that such induction chemotherapy–guided organ-preservation protocols can achieve survival rates comparable to upfront surgery while preserving the larynx in a substantial proportion of patients [45]. For instance, the EORTC-24891 trial in hypopharyngeal SCC reported no significant difference in overall survival between patients receiving induction cisplatin-5-fluorouracil (FU) followed by radiotherapy, in responders, and those undergoing immediate surgery (10-year OS ~ 13% in both arms), but more than half of the long-term survivors in the induction arm retained a functional larynx [46]. In parallel, advances have been made in esophageal SCC management, including tumors at the cervical esophagus which historically mandate PLE for surgical management. Randomized trials and meta-analyses in esophageal SCC have firmly established neoadjuvant therapy as standard of care for locally advanced disease. Both neoadjuvant CRT and neoadjuvant chemotherapy alone have shown significant improvements in survival compared to surgery alone [47,48]. For instance, using combined modality therapy, the CROSS trial (Chemoradiotherapy for Oesophageal Cancer Followed by Surgery) demonstrated that the median survival for the SCC subset was 81.6 months with neoadjuvant CRT versus 21.1 months with surgery alone. Such evidence has made neoadjuvant CRT followed by surgery a standard approach for resectable esophageal SCC in many guidelines [49]. Moreover, a meta-analysis by Sjoquist et al. demonstrated a 23% reduction in mortality risk with neoadjuvant CRT for esophageal cancer compared to surgery alone, and this benefit is presumed to extend to cervical tumors [50]. At the same time, research in East Asia has explored intensive neoadjuvant chemotherapy-only regimens. The recent JCOG 1109 (NExT) trial in Japan compared neoadjuvant doublet chemotherapy, neoadjuvant triplet chemotherapy, and neoadjuvant chemoradiation in esophageal SCC. That phase III study found that triplet chemotherapy-cisplatin, 5-FU, and docetaxel (TPF) before surgery yielded a significant overall survival improvement over the standard doublet (cisplatin/5-FU- whereas adding moderate-dose radiotherapy to the doublet did not significantly increase survival beyond chemotherapy alone. These findings establish that an organ-preserving approach can be highly effective in selected SCC, without necessarily compromising oncologic outcomes, while they highlight the importance of tailoring neoadjuvant treatment intensity to tumor biology and patient fitness [51]. Historically, this paradigm was shaped by landmark laryngeal and hypopharyngeal preservation trials. The VA Laryngeal Cancer Study established induction cisplatin/5-fluorouracil (PF) followed by radiotherapy in responders as a feasible larynx-preserving alternative to upfront surgery in selected patients with advanced laryngeal cancer [52]. EORTC 24891 extended this approach to hypopharyngeal and lateral epilaryngeal SCC, demonstrating comparable long-term survival between induction chemotherapy-based preservation and surgery, although survival remained poor in both arms [53]. RTOG 91 − 11 subsequently established concurrent cisplatin-based chemoradiotherapy as a highly effective organ-preservation strategy, improving laryngeal preservation and locoregional control compared with induction chemotherapy followed by radiotherapy or radiotherapy alone [54]. The addition of docetaxel further refined induction regimens: TAX 323/EORTC 24971 and TAX 324 demonstrated the superiority of docetaxel, cisplatin, and 5-fluorouracil (TPF) over cisplatin and 5-fluorouracil (PF) in locally advanced SCC of the head and neck, while GORTEC 2000-01 confirmed improved larynx preservation and larynx dysfunction-free survival with TPF compared with PF in laryngeal and hypopharyngeal preservation protocols [55, 56].

More recently, immune checkpoint inhibition has begun to reshape curative-intent multimodal therapy. KEYNOTE-689 demonstrated that perioperative pembrolizumab, administered preoperatively and continued postoperatively with risk-adapted adjuvant therapy, significantly improved event-free survival in resectable locally advanced HNSCC without reducing surgical completion rates. Exploratory analyses also suggested improved distant metastasis-free survival, supporting a potential role for perioperative immunotherapy in systemic disease control and neoadjuvant downstaging [57]. These findings led to FDA approval in June 2025 for neoadjuvant and adjuvant pembrolizumab in adults with resectable locally advanced PD-L1-positive HNSCC [58]. In the postoperative setting, NIVOPOSTOP/GORTEC 2018-01 further demonstrated that adding nivolumab to adjuvant cisplatin-based chemoradiotherapy significantly improved disease-free survival in resected high-risk locally advanced HNSCC [59]. Although these data expand the therapeutic landscape, immunotherapy has not yet been established as a definitive organ-preserving substitute for PLE in tumors requiring en bloc pharyngolaryngoesophageal clearance.

Table 1 below summarizes the current regional guidelines (Asia/Japan, United States of America (USA), Europe) for SCC of the hypopharynx/larynx and cervical esophagus – tumors often requiring PLE if managed surgically – including also recommended neoadjuvant therapy, definitive chemoradiation approaches as well as concise surveillance protocols [50, 60, 61].

**Table 1:** Regional guidelines of SCC of Hypopharynx, Larynx and Cervical Esophagus [50, 60, 61]

Region	Chemotherapy	Radiotherapy	Surgery	Surveillance
Japan (Asia)	Neoadjuvant DCF (cisplatin, 5-FU, docetaxel) preferred; replaces CF doublet (JCOG1109).	Definitive CRT for cervical esophagus SCC; preoperative CRT occasionally to aid larynx preservation.	Esophagectomy with larynx preservation if possible; PLE if tumor involves larynx. Salvage surgery after CRT in case of recurrence.	Endoscopy q2–3 mo (year 1), q4–6 mo (year 2), annually ≥ 5 yrs. CT ~ 3× first year, then annually.
United States of America (USA)	Neoadjuvant CRT (CROSS: carboplatin/paclitaxel + 41.4 Gy) standard for thoracic SCC.	Definitive CRT (60–66 Gy + cisplatin/5-FU) for cervical esophagus; 70 Gy CRT for larynx/hypopharynx organ preservation.	Esophagectomy after neoadjuvant CRT for thoracic SCC. PLE avoided upfront for cervical esophagus; reserved for salvage.	H&N exam q1–3 mo (year 1), q2–6 mo (year 2), q4–8 mo (years 3–5), annually. Imaging baseline 3–6 mo; TSH q6–12 mo; annual chest CT if smoker.
Europe	Neoadjuvant CRT (CROSS-like regimen) standard; perioperative chemotherapy not favored for SCC.	Definitive CRT for cervical esophagus SCC (preferred); CRT for larynx/hypopharynx organ preservation.	Esophagectomy after CRT for thoracic SCC. PLE avoided upfront for cervical esophagus; reserved for salvage.	Clinical exam ~ 5× first year, ~ 3×/yr by year 5. Endoscopy annually ≥ 5 yrs. CT 3× first year, then annually. Screen for second primaries.

It is worth noting that, the introduction of neoadjuvant therapy has transformed the treatment algorithm for advanced SCC that may require PLE. When applied thoughtfully, it allows clinicians to personalize treatment. By combining systemic and local treatments in a strategic sequence, we can maximize tumor control, improve cure rates, and in many cases achieve cure with preservation of vital functions – a true advancement in the multidisciplinary management of these challenging cancers [49,62].

Within contemporary multimodal algorithms for SCC of the hypopharynx, larynx, and cervical esophagus, neoadjuvant therapy functions both as a biologic selection tool for organ-preservation protocols and as a means of tumor downstaging prior to definitive surgery, including PLE when indicated. Building on this neoadjuvant setting, immunotherapy is a cutting-edge investigational strategy for resectable head and neck cancers, including those that might eventually require PLE [63]. The advent of immune checkpoint inhibitors (ICIs)– particularly anti-PD-1 and anti-PD-L1 antibodies – has transformed the course treatment in the recurrent/metastatic setting and is now making inroads into earlier stages of disease. Early-phase studies have shown feasibility, encouraging pathological responses, and no consistent signal for surgical delay. A recent systematic review and meta-analysis found that neoadjuvant immunotherapy in resectable SCC of the head and neck is feasible and well-tolerated, with encouraging pathologic response rates and no significant surgical delays [64]. For instance, neoadjuvant pembrolizumab monotherapy in stage III/IVA SCC of the head and neck yielded major pathologic responses in a subset of patients without preventing timely surgery [59]. Incorporating immunotherapy with induction chemotherapy is also under active study; preliminary results suggest higher response rates than chemotherapy alone [44]. In esophageal cancer, immunotherapy has already entered the curative-intent paradigm in the adjuvant setting. The Check Mate 577 trial (2021) demonstrated that adjuvant nivolumab (anti-PD-1 antibody) given for one year after chemoradiation and surgery significantly improved disease-free survival in patients with resected esophageal or gastroesophageal junction cancer. While the majority of that trial’s patients had distal esophageal or junctional tumors, the benefit is believed to extend to cervical esophageal SCC as well. Based on this, for a patient undergoing PLE for esophageal cancer or even combined hypopharyngeal-esophageal cancer, who also had prior neoadjuvant chemoradiotherapy, adjuvant immunotherapy with nivolumab is a new standard if residual disease is found pathologically. This approach has shown a doubling of median disease-free survival (22.4 months with nivolumab vs. 11.0 months with placebo), and emerging data suggests a trend toward improved overall survival as well [65]. Furthermore, trials in China such as ESCORT-NEO have explored neoadjuvant chemo-immunotherapy in esophageal SCC. Early analyses reported markedly high pathologic complete response rates and improved 3-year survival (92% vs. 80% in a retrospective comparison) when an anti-PD-1 agent (e.g. camrelizumab) was added to neoadjuvant chemotherapy [66]. Importantly, PALACE-1 evaluated preoperative pembrolizumab combined with chemoradiotherapy for locally advanced, resectable ESCC and demonstrated feasibility, no treatment-related surgical delay, and a pathological complete response rate of 55.6% among resected patients, supporting further investigation of pembrolizumab-based immunochemoradiotherapy in this setting. Subsequent PALACE-2 was designed as a multicenter study to further evaluate the efficacy and safety of this strategy in locally advanced resectable ESCC [67]. Another pilot study combining chemotherapy, radiotherapy, and the PD-1 inhibitor sintilimab before surgery showed a significantly higher pathological response rate than historical controls with CRT alone [68]. Given these developments, one can envision a multimodal protocol for an advanced hypopharyngeal or cervical esophageal carcinoma in the near future that includes chemo-immunotherapy induction to maximize tumor reduction and immune activation, followed by PLE for local control, and possibly further immunotherapy postoperatively [34, 69]. Also, the integration of immunotherapy into neoadjuvant regimens is almost poised to refine patient selection and optimize outcomes for both organ-preserving approaches and PLE. However, this approach needs validation in clinical trials. At present, immunotherapy is primarily utilized in the recurrent/metastatic setting (e.g. pembrolizumab or nivolumab for unresectable or metastatic HNSCC, and for metastatic esophageal SCC) and as adjuvant therapy after resection in esophageal cancer. Its neoadjuvant use remains investigational but highly promising [70].

The potential benefits of chemotherapy, chemoradiotherapy, and immunotherapy must be balanced against their toxicity profiles. Induction chemotherapy, particularly TPF-based regimens, is associated with substantial hematologic and mucosal toxicity, including grade 3–4 neutropenia, febrile neutropenia, stomatitis, dysphagia, nausea, vomiting, and infection, while chemoradiotherapy may cause acute mucositis, dermatitis, dysphagia, aspiration risk, feeding-tube dependence, and late fibrosis or strictures. Immune checkpoint inhibitors are generally better tolerated than cytotoxic chemotherapy but may cause immune-mediated adverse events, including pneumonitis, colitis, hepatitis, endocrinopathies, nephritis, and dermatologic toxicity; therefore, treatment intensification should be individualized according to expected oncologic benefit, comorbidity, nutritional status, baseline swallowing function, and surgical candidacy [59].

**Fig. 1:**
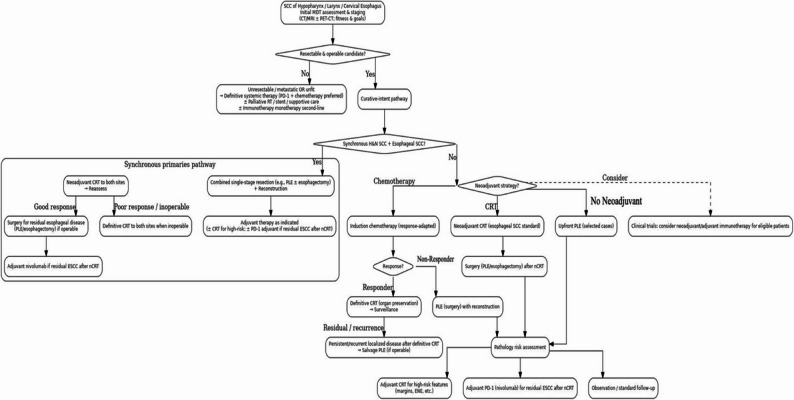
Multimodal treatment algorithm for SCC of the hypopharynx, larynx, and cervical esophagus [19, 20]

In summary, the treatment strategies surrounding PLE are firmly multimodal (Fig. 1) [34, 35]. The current trend is toward maximizing non-surgical therapy either before surgery or instead of surgery when feasible, and reserving PLE for cases where such approaches are insufficient. Neoadjuvant chemotherapy has shown value in improving organ preservation rates and possibly outcomes, while immunotherapy is an emerging powerful adjunct–already standard in adjuvant therapy for esophageal cancer [71]. Ultimately, the optimal sequencing of therapy is individualized; some patients will be best served by immediate surgery, whereas others can benefit from tumor shrinkage and systemic control through neoadjuvant therapy as well as immunotherapy before undergoing PLE. A multidisciplinary tumor board review is essential to tailor the plan, and whenever possible, patients should be enrolled in clinical trials exploring these evolving paradigms.

## Conclusions

In conclusion, while PLE is a well-established, albeit aggressive, procedure, its role is being continually refined in the context of advancing non-surgical therapies. The future role of PLE will be shaped by advances in patient selection, systemic therapy and surgical innovation. Efforts are underway to refine selection through molecular profiling, imaging, and response-adapted strategies, which may help identify patients most likely to benefit from surgery versus organ-preserving approaches. Immunotherapy is rapidly transforming the treatment landscape, poised to establish new standards of perioperative care. The future likely holds a more nuanced integration of surgery with targeted therapies like immunotherapy, improved safety through minimally invasive techniques and better tissue healing, and hopefully better survival and functional outcomes as a result. Finally, comparative effectiveness research will be critical to define the relative value of surgery versus definitive CRT in specific subsets of patients, particularly as systemic therapies evolve. Through these incremental improvements and innovations, the hope is that we can improve the cure rates of these challenging cancers while minimizing the impact of treatment on patients’ lives. These developments underscore a future in which PLE is more personalized, safer, and integrated within multimodal treatment paradigms.

## Supplementary Information

Supplementary Material 1.

## Data Availability

No datasets were generated or analysed during the current study.

## Abbreviations

ALT: Anterolateral thigh
CRT: Chemoradiotherapy
DFS: Disease-free survival
ESCC: Esophageal squamous cell carcinoma
FU: Fluorouracil
HNSCC: Head and neck squamous cell carcinoma
ICI: Immune checkpoint inhibitor
MIPLE: Minimally invasive pharyngolaryngoesophagectomy
OS: Overall survival
PD-1: Programmed cell death protein 1
PD-L1: Programmed death-ligand 1
PLE: Pharyngolaryngoesophagectomy
QoL: Quality of life
SCC: Squamous cell carcinoma
TEP: Tracheoesophageal puncture
USA: United States of America
