# Supplementary material for: Bridging the gap: modern pharyngolaryngoesophagectomy techniques and the rise of neoadjuvant/immunotherapy approaches
Source: World J Surg Oncol. 2026 Jul 31;24:319. doi: 10.1186/s12957-026-04519-9 (PMC13430872; doi:10.1186/s12957-026-04519-9)
Supplement: Supplementary file 1 — Supplementary Material 1. [file 12957_2026_4519_MOESM1_ESM.pdf]

REVISEDDRAFT_UPLOAD.docx

Karolinska Institute

Document Details

Submission ID

trn:oid:::3117:588131496

Submission Date

May 8, 2026, 11:46 PM GMT+2

Download Date

May 8, 2026, 11:48 PM GMT+2

File Name

REVISEDDRAFT_UPLOAD.docx

File Size

363.5 KB

21 Pages

6,318 Words

40,380 Characters

9% Overall Similarity

The combined total of all matches, including overlapping sources, for each database.

Filtered from the Report

- Bibliography
- Small Matches (less than 9 words)

Match Groups

24 Not Cited or Quoted 8%
Matches with neither in-text citation nor quotation marks

8 Missing Quotations 2%
Matches that are still very similar to source material

0 Missing Citation 0%
Matches that have quotation marks, but no in-text citation

0 Cited and Quoted 0%
Matches with in-text citation present, but no quotation marks

Top Sources

8% Internet sources
8% Publications
0% Submitted works (Student Papers)

Integrity Flags

0 Integrity Flags for Review

No suspicious text manipulations found.

Our system's algorithms look deeply at a document for any inconsistencies that would set it apart from a normal submission. If we notice something strange, we flag it for you to review.

A Flag is not necessarily an indicator of a problem. However, we'd recommend you focus your attention there for further review.

Match Groups

- **24** Not Cited or Quoted 8%
  Matches with neither in-text citation nor quotation marks
- **8** Missing Quotations 2%
  Matches that are still very similar to source material
- **0** Missing Citation 0%
  Matches that have quotation marks, but no in-text citation
- **0** Cited and Quoted 0%
  Matches with in-text citation present, but no quotation marks

Top Sources

8% Internet sources
8% Publications
0% Submitted works (Student Papers)

Top Sources

The sources with the highest number of matches within the submission. Overlapping sources will not be displayed.

1	Internet	www.researchsquare.com	2%
2	Internet	cancerneo.ru	1%
3	Internet	pubmed.ncbi.nlm.nih.gov	<1%
4	Internet	www.preprints.org	<1%
5	Internet	www.medrxiv.org	<1%
6	Internet	www.nice.org.uk	<1%
7	Publication	"Esophageal Adenocarcinoma", Springer Science and Business Media LLC, 2018	<1%
8	Publication	Lefebvre, J.- L., G. Andry, D. Chevalier, B. Luboinski, L. Collette, L. Traissac, D. de R...	<1%
9	Publication	Xizhao Sui, Duoji Danzeng, Ping Ni, Jiayi Geng, Pingcuo Gesang, Renqing Zhaxi, Yu...	<1%
10	Internet	link.springer.com	<1%

11 Publication

Efstathiou, J.A.. "Cardiovascular Mortality and Duration of Androgen Deprivation ... <1%

12 Publication

Arlene A. Forastiere, Nofisat Ismaila, Jan S. Lewin, Cherie Ann Nathan et al. "Use o... <1%

13 Internet

blog.oncoprescribe.com <1%

14 Internet

journals.publisso.de <1%

15 Internet

www.researchgate.net <1%

16 Publication

"Risk Factors and Therapy of Esophagus Cancer", Springer Science and Business ... <1%

17 Internet

ebin.pub <1%

18 Internet

referencecitationanalysis.com <1%

19 Internet

www.ijmpo.org <1%

20 Publication

"Cancer of the Oral Cavity, Pharynx and Larynx", Springer Nature, 2016 <1%

21 Publication

Jun Li, Tao Xu, Binbin Pan, Long Duo, Yue Cao, Fengjun Cao, Yunyan Tai. "Impact ... <1%

22 Publication

Zhigang Wu, Qiang Zheng, Haiquan Chen, Jiaqing Xiang et al. "Efficacy and safety ... <1%

23 Internet

azpdf.tips <1%

24 Internet

healthdocbox.com <1%

iThenticate Page 4 of 26 - Integrity Overview Submission ID trn:oid:::3117:588131496

25 Internet

ro-journal.biomedcentral.com <1%

26 Internet

spj.science.org <1%

27 Internet

www.mdpi.com <1%

28 Internet

www.medilib.ir <1%

Bridging the Gap: Modern Pharyngolaryngoesophagectomy Techniques and the Rise of Neoadjuvant/Immunotherapy Approaches

Freideriki Nteka MD[1], Tania Triantafyllou MD, PhD[2], Ioannis Rouvelas MD, PhD[3], Ioannis Gkoutziotis MD, PhD[4], Panagiotis Sakarellos MD, PhD[1], Maria Tolia MD, PhD[5], Ioannis Karavokyros MD, PhD[1], Konstantinos Mpallas MD, PhD[4], Dimitrios Schizas MD, PhD[1]

3 [1] First Department of Surgery, National and Kapodistrian University of Athens, Laikon General Hospital, Athens, Greece

[2] First Propaedeutic Department of Surgery, National and Kapodistrian University of Athens, Hippocration General Hospital, Athens, Greece 2

[3] Department of Upper Abdominal Surgery, Center for Digestive Diseases, Karolinska University Hospital, Huddinge, and the Division of Surgery and Oncology, Department of Clinical Science, Intervention and Technology (CLINTEC), Karolinska Institutet, Stockholm, Sweden

18 [4] Fifth Department of Surgery, Aristotle University of Thessaloniki, Hippocration Hospital, Thessaloniki, Greece

[5] Department of Radiotherapy, School of Medicine, University of Crete, Heraklion, Greece

2 **<u>Corresponding author</u>:** Ioannis Rouvelas, Department of Upper Abdominal Surgery, Center for Digestive Diseases, Karolinska University Hospital, Huddinge, and the Division of Surgery and Oncology, Department of Clinical Science, Intervention and Technology (CLINTEC), Karolinska Institutet, 141 86 Stockholm, Sweden

email: ioannis.rouvelas@ki.se

Abstract

Background: The purpose of this review is to outline the evolution and changing role of pharyngolaryngoesophagectomy (PLE) in the organ-preservation era, and to review advances, including minimally invasive techniques, neoadjuvant therapy, and immunotherapy, and their impact on outcomes in advanced laryngeal, hypopharyngeal, and cervical esophageal cancers.

Main body: A narrative review of the literature from 2000 to the present was performed, focusing on studies of surgical innovations, oncologic outcomes, quality of life, and combined treatment strategies in PLE. Historically, PLE was associated with high morbidity and mortality. However, advances in surgical techniques, reconstructive methods, and perioperative care have significantly improved its safety and functional outcomes. Minimally invasive and hybrid PLE approaches reduce pulmonary complications and speed recovery without compromising oncologic outcomes. Chemoradiation has become the first-line treatment for many advanced cases, with PLE now reserved for selected cervical esophageal tumors or persistent disease after radiation. Neoadjuvant chemotherapy or chemoradiation can shrink tumors, improve operability, and sometimes allow organ preservation. Immunotherapy before surgery improves pathological response rates and disease-free survival. Recent series report 5-year survival of 20–40% after PLE, and most patients regain swallowing and voice function with rehabilitation.

Conclusion: In the organ-preservation era, PLE remains important for certain advanced or treatment-resistant cases. Its role is defined by a multidisciplinary, personalized approach and limited to cases where surgery is essential, often combined with systemic therapy. Ongoing advances in patient selection, minimally invasive techniques, and immunotherapy are expected to further refine PLE's role and improve outcomes.

Keywords: Pharyngolaryngoesophagectomy, Neoadjuvant therapy, Immunotherapy, Morbidity, Mortality

Background

14 This narrative review examines the contemporary role of pharyngolaryngoesophagectomy in the management of advanced squamous cell carcinoma of the larynx, hypopharynx, and cervical esophagus. In the organ-preservation era, its role has become increasingly selective and is now more clearly defined within multidisciplinary treatment algorithms, while advances in reconstruction, perioperative care, and minimally invasive surgery have improved its safety and functional outcomes. In parallel, neoadjuvant therapy and immunotherapy are reshaping treatment strategies and may further refine the role of surgery in this setting.

Main Text

Introduction

19 Squamous cell carcinoma (SCC) is the predominant histologic subtype of malignancies affecting the hypopharynx, larynx, and cervical esophagus. These tumors share common etiologic factors, most notably tobacco and alcohol exposure, and are often characterized by aggressive local invasion, submucosal spread, and a high propensity for regional nodal metastasis [1]. Advanced-stage SCC in these sites frequently extends across anatomical boundaries, necessitating en bloc resection for oncologic clearance. Despite advances in organ-preservation protocols, SCC involving the pharyngolaryngeal-esophageal axis continues to present a therapeutic challenge, and pharyngolaryngoesophagectomy (PLE) remains an important consideration in selected cases where non-surgical approaches are unlikely to achieve durable disease control. In particular, esophageal squamous cell carcinoma (ESCC) of the cervical esophagus represents a distinct clinical entity, comprising a small minority of all esophageal cancers but carrying a poor prognosis. Due to its anatomic proximity to the larynx and hypopharynx, curative resection almost invariably requires PLE, and while definitive chemoradiotherapy is often employed with organ-preservation intent, surgical resection continues to provide an essential option for selected patients with localized or treatment-refractory disease [2]. PLE is an extensive surgical procedure involving en bloc resection of the pharynx, larynx, and a portion or entirety of the esophagus. It is typically reserved for selected advanced upper aerodigestive tract cancers that expand to these anatomical regions. Historically, it is considered a procedure of last resort due to its high morbidity and functional impairment (loss of natural voice and normal swallowing). PLE
23 remains an important tool in the multidisciplinary management of certain head and neck and proximal esophageal
3 malignancies [3]. In the modern era of organ preservation – wherein many laryngeal and hypopharyngeal cancers

are treated with definitive chemoradiation to avoid total laryngectomy – the role of PLE has evolved. Contemporary indications are more narrowly defined, often centering on tumors where organ-preserving therapies are unlikely to achieve cure or acceptable function, for instance, tumors recurring after radiation. Through recent decades, significant advances have been seen in surgical technique, reconstruction, and adjuvant therapies, which have collectively improved the safety profile of PLE and expanded reconstructive options. Nonetheless, PLE remains a formidable intervention, and its outcomes continue to be closely scrutinized against non-surgical approaches such as combined chemoradiotherapy [4].

Herein, this narrative review provides a comprehensive overview of PLE for SCC involving the larynx, hypopharynx, and cervical esophagus, with emphasis on the contemporary literature from 2000 onward. It synthesizes the historical development of PLE, delineates current indications in both primary and salvage settings, and expands on modern operative and reconstructive refinements, including advances in perioperative care, microvascular and visceral conduit reconstruction, minimally invasive and hybrid approaches, highlighting postoperative outcomes, complication profiles, functional rehabilitation, long-term survival as well as patient-centered quality of life. Finally, the growing role of multimodal management, with emphasis on neoadjuvant systemic therapy and the emerging integration of immunotherapy, in optimizing patient selection, improving functional outcomes, and refining the role of PLE, is discussed.

Historical Background

The concept of en bloc resection of the larynx, pharynx, and esophagus for cancer has its roots in the late 19th and mid-20th centuries. Early attempts at pharyngoesophageal resection were fraught with technical challenges and prohibitive mortality. It was not until the mid-20th century that PLE became a viable even though high-risk option, mostly due to improvements in anesthesia, surgical techniques, and perioperative care. Total PLE as a formal procedure was introduced around 1960, initially accompanied by very high perioperative mortality rates exceeding 30%. In these early decades, two-stage reconstructions such as the Wookey two-step skin flap technique and other regional flaps were employed for pharyngoesophageal defects. By the 1960s–1990s, visceral transposition techniques became standard: gastric pull-up (pharyngogastric anastomosis) and colon interposition were favored to replace the resected esophagus and pharynx. Concurrently, pedicled regional flaps (e.g. deltopectoral and pectoralis major flaps) were used to assist in closure of large neck wounds or reinforce repairs. The late 1980s marked a revolution in reconstruction with the advent of microvascular free tissue transfer. Fasciocutaneous free flaps such as the radial forearm free flap and the anterolateral thigh (ALT) flap began to be used for

pharyngoesophageal reconstruction, offering well-vascularized tissue to reconstruct partial pharyngeal or short-segment circumferential esophageal defects. Free jejunal interposition grafts also emerged as a figurative option for esophageal replacement after PLE, first described by Seidenberg in 1959 and increasingly utilized due to beneficial outcomes compared to earlier methods. These advancements in reconstructive strategies provided a tailored approach that dramatically decreased the morbidity of reconstruction, while they enhanced the success rates of restoring alimentary continuity and airway safety [5]. Notably, functional outcomes after PLE have improved considerably due to modern reconstructive techniques. Modern outcomes demonstrate that the vast majority of patients resume oral feeding within a few months, with over 90% progressing to a soft or complete diet. Voice rehabilitation is also possible, most commonly through tracheoesophageal puncture (TEP) or alternatively with electrolarynx devices, enabling the majority of patients to regain functional communication. By the 21st century, mortality and complication rates for PLE had noticeably improved. Reports from the 1990s noted that perioperative mortality had fallen below 5–10% in high-volume centers, and anastomotic leak rates, once as high as 20–30%, had decreased to under 10%. The procedure that was once deemed almost prohibitive began to be performed with acceptable safety [6]. The historical evolution of PLE is also intertwined with changes in nonsurgical therapy. The 1990s and 2000s organ-preservation protocols (concurrent chemoradiation and induction chemotherapy strategies) became standard for many advanced laryngeal/hypopharyngeal cancers, thereby reducing the frequency of upfront PLE. The role of PLE shifted more towards to salvage surgery and thus PLE transformed from a high-risk, ultimate operation to a more refined procedure supported by improved reconstruction alternatives as well as perioperative care. This historical evolution informs contemporary practice, in which PLE is reserved for carefully selected patients within a multidisciplinary framework [7].

Technical Steps and Evolving Strategies in PLE

In the traditional open approach, PLE is performed via combined cervical and abdominal incisions- with or without a thoracic incision- to access all involved regions. The operation entails en bloc removal of the larynx with partial pharyngectomy at the cervical level, combined with a complete esophagectomy. Mobilization of the cervical and upper thoracic esophagus is performed through the cervical incision, while the distal esophagus and stomach are dissected via an abdominal approach. Following resection, digestive continuity is typically restored by constructing a cervical anastomosis. Given the magnitude of this surgery, open PLE has traditionally carried significant morbidity and poses complex reconstructive challenges. Immediate reconstruction is most commonly achieved by utilizing a gastric conduit (gastric pull-up) owing to its dependable vascularity and adequate length.

In select cases, alternative conduits such as a colon interposition or a free jejunal graft are employed, often employing microvascular augmentation in order to enhance perfusion and reduce the risk of graft necrosis or anastomotic leak [8].

Over the past two decades, PLE has evolved through refinements in both access and reconstruction through minimally invasive approaches, aimed at reducing complications and improving function. Building on traditional open cervical resection combined with thoracoscopic and laparoscopic techniques in esophageal surgery, minimally invasive PLE (MIPLE) has emerged as a feasible alternative. Early reports demonstrated the technical feasibility of combining thoracoscopic esophagectomy and laparoscopic gastric mobilization with standard cervical resection, showing that MIPLE can be performed safely in highly selected patients [9]. Comparative analyses indicate that, although MIPLE does not eliminate the inherent complexity of the operation, it may reduce pulmonary morbidity, postoperative pain, and length of hospitalization while achieving oncologic outcomes comparable to those of open surgery. However, the open cervical phase of the surgery remains the major source of complications, including pharyngogastric leaks and tracheal stump compromise, thereby limiting the overall reduction in morbidity. Herein, meticulous surgical technique, preservation of vascular supply, and selective application of hybrid approaches, are essential. Currently, MIPLE is generally reserved for selected tumors requiring combined resection of the larynx, pharynx, and cervical or upper thoracic esophagus, predominantly in high-volume centers with expertise in both head and neck and esophageal surgery [10]. Moreover, emerging reports suggest that robot-assisted techniques may further refine minimally invasive esophagectomy as well as MIPLE by enhancing surgical dexterity and visualization, although their adoption remains limited and largely confined to specialized centers. In other words, MIPLE represents an evolution rather than a replacement of the traditional open technique, contributing to improved perioperative outcomes while preserving oncologic principles in the multidisciplinary management of advanced hypopharyngeal and cervical esophageal cancers within multidisciplinary care pathways [11].

Indications for PLE in the Organ Preservation Era

In the era of organ preservation, most locally advanced laryngeal and hypopharyngeal squamous cell carcinomas are treated initially with combined chemotherapy and radiation or radiation alone in some cases with the intent of avoiding total laryngectomy. However, PLE retains an important role when tumors extend beyond the larynx/hypopharynx into adjacent structures that make organ-preserving approaches less effective [12]. A classic

indication is a hypopharyngeal carcinoma that invades the cervical esophagus. Such tumors are typically staged as very advanced (T4 disease, often stage IVB) and historically had extremely poor outcomes. In these cases, definitive chemoradiotherapy may have a low likelihood of achieving complete response and carries a risk of complications, for example tumor-associated esophageal perforation or fistula creation during radiation. Thus, PLE may be recommended for a chance to achieve clear margins and oncologic control. Indeed, current clinical guidelines note that for hypopharyngeal cancers with esophageal extension or extensive cartilage destruction (T4 stage), surgery-based therapy is a reasonable or even preferred option, as organ preservation yields suboptimal results in this subset [13]. PLE is, also, indicated for advanced hypopharyngeal cancers that expand submucosally into the upper esophagus, where a total laryngopharyngectomy combined with esophagectomy is required to achieve clear margins. Similarly, laryngeal carcinomas which extend to the post-cricoid area or esophageal inlet may necessitate PLE, especially if they are not expected to respond adequately to chemoradiation or if significant tumor-associated dysphagia precludes waiting for radiation effects. Moreover, cancers of the cervical esophagus (typically defined as tumors with epicenter within 5 cm distal to the cricopharyngeus) are a distinct indication for PLE. These tumors are relatively rare (<5% of esophageal cancers) but pose a unique challenge. Surgical resection of a cervical esophageal carcinoma almost always requires removal of the adjacent larynx and pharynx to achieve an adequate proximal margin, essentially mandating a PLE for oncologic resection. Current guidelines recommend definitive chemoradiotherapy as first-line treatment for cervical esophageal squamous cell carcinoma, given its potential for laryngeal preservation and survival comparable to surgery. Nevertheless, surgery remains indicated in selected circumstances—namely when definitive chemoradiotherapy is contraindicated or has failed; when a resectable tumor is causing substantial symptoms or as a primary option for bulky or extensive disease- unlikely to be controlled by radiotherapy as well as in patients unfit for high-dose radiotherapy. PLE may, also, be indicated for synchronous head and neck and esophageal squamous cell carcinomas, as it enables en bloc resection of both malignancies in a single procedure and can provide favorable survival outcomes in carefully selected patients despite its high morbidity [14]. Another key indication for PLE is salvage surgery in the setting of recurrent or persistent disease after primary chemoradiotherapy. Salvage PLE is indicated particularly when the recurrence involves the cervical esophagus or is diffuse in the pharyngolaryngeal region such that a less extensive resection (e.g. total laryngectomy alone) would not completely excise the disease. While organ-preservation protocols are commonly applied in laryngeal and hypopharyngeal cancers, persistent or recurrent disease remains a clinical challenge, and prior radiotherapy may compromise subsequent surgical management because of fibrosis and impaired healing. PLE can be a valid option with curative intent, removing the irradiated larynx/pharynx and

esophagus en bloc. It should be noted that salvage PLE carries higher risks of complications (due to irradiated tissue) and often requires flap reconstruction to ensure healing, but it can provide long-term disease control in selected patients [15].

The decision to undertake PLE is determined through multidisciplinary deliberation, balancing the potential benefits of non-surgical modalities against the risks inherent to extensive surgery. PLE is generally reserved for: (1) tumors with extension to the cervical esophagus or other situations where chemoradiation is unlikely to succeed, (2) synchronous head and neck and esophageal cancers amenable to single-stage resection, and (3) salvage of locally recurrent disease after failed chemoradiotherapy. As organ-preserving treatments improve, the indications for primary PLE have narrowed, but PLE remains indispensable for certain advanced cases where it offers the best curative course [16].

Postoperative Survival and Quality of Life

Nowadays, PLE operative mortality has declined to acceptable levels, and a meaningful proportion of patients has achieved prolonged survival. For example, a series of 208 patients undergoing PLE with gastric pull-up reconstruction reported no intraoperative deaths and only four in-hospital fatalities (~2%). Likewise, reports of MIPLE have documented zero 30-day mortality [17]. Although operative mortality after PLE is now generally in the low-single-digit range, morbidity remains high. Complication rates typically range from 40% to 69% and the risk of leaks or fistulas (5-10%), and respiratory issues after PLE are emphasized, while preoperative radiotherapy and poor patient condition further increase complication rates [18]. Such events predispose to pharyngocutaneous or tracheoesophageal fistulation and commonly delay the resumption of oral feeding. Anastomotic stricture is reported in about 11% of patients, most often after postoperative radiotherapy, and typically necessitates repeated endoscopic dilations. Conduit and wound complications vary by technique, cervical skin-flap necrosis was the most frequent event (up to 34%), following wound infection in 4% with occasional reoperation. Respiratory morbidity remains prominent, with pneumonia or respiratory failure in approximately 10–15% of patients [19].

Long-term survival following PLE remains limited, largely reflecting the advanced stage and aggressive biology of the tumors that necessitate this procedure. Most contemporary series report 5-year overall survival (OS) in the range of 20–40%, with median survival typically between 17 and 30 months [20]. Survival outcomes after PLE are broadly comparable to those achieved with definitive chemoradiotherapy for cervical esophageal and advanced

hypopharyngeal cancers (5-year OS ~20–35%), yet non-surgical approaches are often favored for their quality-of-life benefits, while surgery remains justified in selected patients likely to achieve durable control [21].

Despite these challenges, many long-term survivors report acceptable functional status. Overall quality of life is generally favorable, with patients typically reporting better outcomes in domains related to swallowing and nutritional independence. Post-gastrectomy sequelae such as esophageal reflux and dumping syndrome may occur after gastric pull-up; however, most patients adapt with dietary modifications [22]. Notably, communication is restored through alternative methods like esophageal speech, electrolarynx, or tracheoesophageal puncture [23].

Quality of life is crucially contingent upon the avoidance of major complications. Anastomotic leaks typically necessitate weeks of non-oral (enteral) feeding, intensive wound care, and occasionally reoperation, resulting in nutritional compromise and delayed initiation of speech and swallowing therapy [24]. Adjuvant radiotherapy—commonly required in advanced disease—further elevates stricture risk and can induce chronic edema and fibrosis, thereby exacerbating dysphagia. Consequently, optimal recovery depends on coordinated multidisciplinary management, including early speech–language pathology involvement, structured swallowing exercises, individualized nutritional support, and comprehensive psychosocial care [25].

Among carefully selected patients, PLE can achieve acceptable long-term function, with most survivors regaining adequate swallowing and nutritional autonomy without permanent enteral support, although communication and social functioning often remain limited. Given that 5-year survival is broadly similar with surgery and definitive chemoradiotherapy (~20–35%), treatment selection should balance expected oncologic control against procedure-related morbidity and functional quality-of-life outcomes [26]. Multidisciplinary support, including speech and swallowing therapy, nutritional guidance and psychosocial services, is essential, and in carefully selected patients, upfront surgery can yield acceptable functional results while avoiding the added morbidity of salvage procedures in irradiated fields [27].

Multimodal Treatment Strategies and the Role of Neoadjuvant Therapy

Treatment of cancers that may require PLE involves a comprehensive multimodal strategy, combining surgical resection with radiotherapy, chemotherapy, and emerging targeted systemic approaches. The optimal therapeutic approach is determined on an individual basis, guided by tumor site, stage, biological behavior, and patient-related variables [3]. In recent years, neoadjuvant therapy has been introduced as part of this multimodal approach,

bringing significant benefits in terms of both survival and functional preservation. Neoadjuvant treatment can consist of chemotherapy alone (induction chemotherapy) or chemoradiotherapy (CRT). Organ-preservation protocols, referring to induction chemotherapy–guided radiotherapy or concurrent CRT, can achieve survival comparable to upfront PLE in advanced laryngeal/hypopharyngeal cancer, even when cervical esophageal extension is present [28]. A response-adapted strategy using induction chemotherapy identifies candidates for definitive CRT, yielding substantial laryngeal and esophageal preservation without increasing severe complications, while triaging non-responders to surgery. Accordingly, induction chemotherapy is employed as a practical selection tool to individualize treatment and maximize functional preservation if oncologically feasible [29]. Beyond its role in organ preservation decision-making, induction chemotherapy is also used with the intent of downstaging tumors prior to surgery and eradicating micrometastatic disease. Induction can sometimes shrink the tumor, potentially converting an unresectable tumor to resectable or simplifying the subsequent surgery [30].

As evidenced by randomized trials and meta-analyses, both neoadjuvant induction chemoradiation and chemotherapy have proven efficacy in squamous cell carcinoma. More specifically, pioneering trials in laryngeal and hypopharyngeal cancers demonstrated that such induction chemotherapy–guided organ-preservation protocols can achieve survival rates comparable to upfront surgery while preserving the larynx in a substantial proportion of patients [31]. For instance, the EORTC-24891 trial in hypopharyngeal SCC reported no significant difference in overall survival between patients receiving induction cisplatin-5-fluorouracil (FU) followed by radiotherapy, in responders, and those undergoing immediate surgery (10-year OS ~13% in both arms), but more than half of the long-term survivors in the induction arm retained a functional larynx [32]. In parallel, advances have been made in esophageal SCC management, including tumors at the cervical esophagus which historically mandate PLE for surgical management. Randomized trials and meta-analyses in esophageal SCC have firmly established neoadjuvant therapy as standard of care for locally advanced disease. Both neoadjuvant CRT and neoadjuvant chemotherapy alone have shown significant improvements in survival compared to surgery alone [33], [34]. For instance, using combined modality therapy, the CROSS trial (Chemoradiotherapy for Oesophageal Cancer Followed by Surgery) demonstrated that the median survival for the SCC subset was 81.6 months with neoadjuvant CRT versus 21.1 months with surgery alone. Such evidence has made neoadjuvant CRT followed by surgery a standard approach for resectable esophageal SCC in many guidelines [35]. Moreover, a meta-analysis by Sjoquist *et al.* demonstrated a 23% reduction in mortality risk with neoadjuvant CRT for esophageal cancer compared to surgery alone, and this benefit is presumed to extend to cervical tumors [36]. At the same time, research in East Asia has explored intensive neoadjuvant chemotherapy-only regimens. The recent JCOG 1109 (NExT) trial in

Japan compared neoadjuvant doublet chemotherapy, neoadjuvant triplet chemotherapy, and neoadjuvant chemoradiation in esophageal SCC. That phase III study found that triplet chemotherapy (cisplatin, 5-FU, and docetaxel) before surgery yielded a significant overall survival improvement over the standard doublet (cisplatin/5-FU), whereas adding moderate-dose radiotherapy to the doublet did not significantly increase survival beyond chemotherapy alone. These findings establish that an organ-preserving approach can be highly effective in SCC, without necessarily compromising oncologic outcomes, while they highlight the importance of tailoring neoadjuvant treatment intensity to tumor biology and patient fitness [37]. Table 1 below summarizes the current regional guidelines (Asia/Japan, United States of America (USA), Europe) for SCC of the hypopharynx/larynx and cervical esophagus – tumors often requiring PLE if managed surgically – including also recommended neoadjuvant therapy, definitive chemoradiation approaches as well as concise surveillance protocols [36,38,39].

Table 1. *Regional guidelines of SCC of Hypopharynx, Larynx and Cervical Esophagus [36,38,39].*

Region	Chemotherapy	Radiotherapy	Surgery	Surveillance
**Japan (Asia)**	Neoadjuvant DCF (cisplatin, 5-FU, docetaxel) preferred; replaces CF doublet (JCOG1109).	Definitive CRT for cervical esophagus SCC; preoperative CRT occasionally to aid larynx preservation.	Esophagectomy with larynx preservation if possible; PLE if tumor involves larynx. Salvage surgery after CRT in case of recurrence.	Endoscopy q2–3 mo (year 1), q4–6 mo (year 2), annually ≥5 yrs. CT ~3× first year, then annually.
**United States of America (USA)**	Neoadjuvant CRT (CROSS: carboplatin/paclitaxel + 41.4 Gy) standard for thoracic SCC.	Definitive CRT (60–66 Gy + cisplatin/5-FU) for cervical esophagus; 70 Gy CRT for larynx/hypopharynx organ preservation.	Esophagectomy after neoadjuvant CRT for thoracic SCC. PLE avoided upfront for cervical esophagus; reserved for salvage.	H&N exam q1–3 mo (year 1), q2–6 mo (year 2), q4–8 mo (years 3–5), annually. Imaging baseline 3–6 mo; TSH q6–12 mo; annual chest CT if smoker.
**Europe**	Neoadjuvant CRT (CROSS-like regimen) standard; perioperative chemotherapy not favored for SCC.	Definitive CRT for cervical esophagus SCC (preferred); CRT for larynx/hypopharynx organ preservation.	Esophagectomy after CRT for thoracic SCC. PLE avoided upfront for cervical esophagus; reserved for salvage.	Clinical exam ~5× first year, ~3×/yr by year 5. Endoscopy annually ≥5 yrs. CT 3× first year, then annually. Screen for second primaries.

22 It is worth noting that, the introduction of neoadjuvant therapy has transformed the treatment algorithm for advanced SCC that may require PLE. When applied thoughtfully, it allows clinicians to personalize treatment. By combining systemic and local treatments in a strategic sequence, we can maximize tumor control, improve cure rates, and in many cases achieve cure with preservation of vital functions – a true advancement in the multidisciplinary management of these challenging cancers [35],[40].

Within contemporary multimodal algorithms for SCC of the hypopharynx, larynx, and cervical esophagus, neoadjuvant therapy functions both as a biologic selection tool for organ-preservation protocols and as a means of tumor downstaging prior to definitive surgery, including PLE when indicated. Building on this neoadjuvant setting, immunotherapy is a cutting-edge investigational strategy for resectable head and neck cancers, including those
5 that might eventually require PLE [41]. The advent of immune checkpoint inhibitors (ICIs)– particularly anti-PD-1 and anti-PD-L1 antibodies – has transformed the course treatment in the recurrent/metastatic setting and is now making inroads into earlier stages of disease. Early-phase studies have shown feasibility, encouraging pathological
17 responses, and no consistent signal for surgical delay. A recent systematic review and meta-analysis found that neoadjuvant immunotherapy in resectable SCC of the head and neck is feasible and well-tolerated, with encouraging pathologic response rates and no significant surgical delays [42]. For instance, neoadjuvant pembrolizumab monotherapy in stage III/IVA SCC of the head and neck yielded major pathologic responses in a subset of patients without preventing timely surgery [43]. Incorporating immunotherapy with induction chemotherapy is also under active study; preliminary results suggest higher response rates than chemotherapy alone [44]. In esophageal cancer, immunotherapy has already entered the curative-intent paradigm in the adjuvant setting. The Check Mate 577 trial (2021) demonstrated that adjuvant nivolumab (anti-PD-1 antibody) given for
9 one year after chemoradiation and surgery significantly improved disease-free survival in patients with resected esophageal or gastroesophageal junction cancer. While the majority of that trial's patients had distal esophageal or junctional tumors, the benefit is believed to extend to cervical esophageal SCC as well. Based on this, for a patient undergoing PLE for esophageal cancer or even combined hypopharyngeal-esophageal cancer, who also had prior neoadjuvant chemoradiotherapy, adjuvant immunotherapy with nivolumab is a new standard if residual
6 disease is found pathologically. This approach has shown a doubling of median disease-free survival (22.4 months with nivolumab vs 11.0 months with placebo), and emerging data suggests a trend toward improved overall survival as well [45]. Furthermore, trials in China such as ESCORT-NEO have explored neoadjuvant chemo-immunotherapy in esophageal SCC. Early analyses reported markedly high pathologic complete response rates and improved 3-year survival (92% vs 80% in a retrospective comparison) when an anti-PD-1 agent (e.g.

camrelizumab) was added to neoadjuvant chemotherapy [46]. Another pilot study combining chemotherapy, radiotherapy, and the PD-1 inhibitor sintilimab before surgery showed a significantly higher pathological response rate than historical controls with CRT alone [47]. Given these developments, one can envision a multimodal protocol for an advanced hypopharyngeal or cervical esophageal carcinoma in the near future that includes chemo-immunotherapy induction to maximize tumor reduction and immune activation, followed by PLE for local control, and possibly further immunotherapy postoperatively [19,48]. Also, the integration of immunotherapy into neoadjuvant regimens is almost poised to refine patient selection and optimize outcomes for both organ-preserving approaches and PLE. However, this approach needs validation in clinical trials. At present, immunotherapy is primarily utilized in the recurrent/metastatic setting (e.g. pembrolizumab or nivolumab for unresectable or metastatic HNSCC, and for metastatic esophageal SCC) and as adjuvant therapy after resection in esophageal cancer. Its neoadjuvant use remains investigational but highly promising [49].

In summary, the treatment strategies surrounding PLE are firmly multimodal (Figure 1) [19,20]. The current trend is toward maximizing non-surgical therapy either before surgery or instead of surgery when feasible, and reserving PLE for cases where such approaches are insufficient. Neoadjuvant chemotherapy has shown value in improving organ preservation rates and possibly outcomes, while immunotherapy is an emerging powerful adjunct–already standard in adjuvant therapy for esophageal cancer [50]. Ultimately, the optimal sequencing of therapy is individualized; some patients will be best served by immediate surgery, whereas others can benefit from tumor shrinkage and systemic control through neoadjuvant therapy as well as immunotherapy before undergoing PLE. A multidisciplinary tumor board review is essential to tailor the plan, and whenever possible, patients should be enrolled in clinical trials exploring these evolving paradigms.

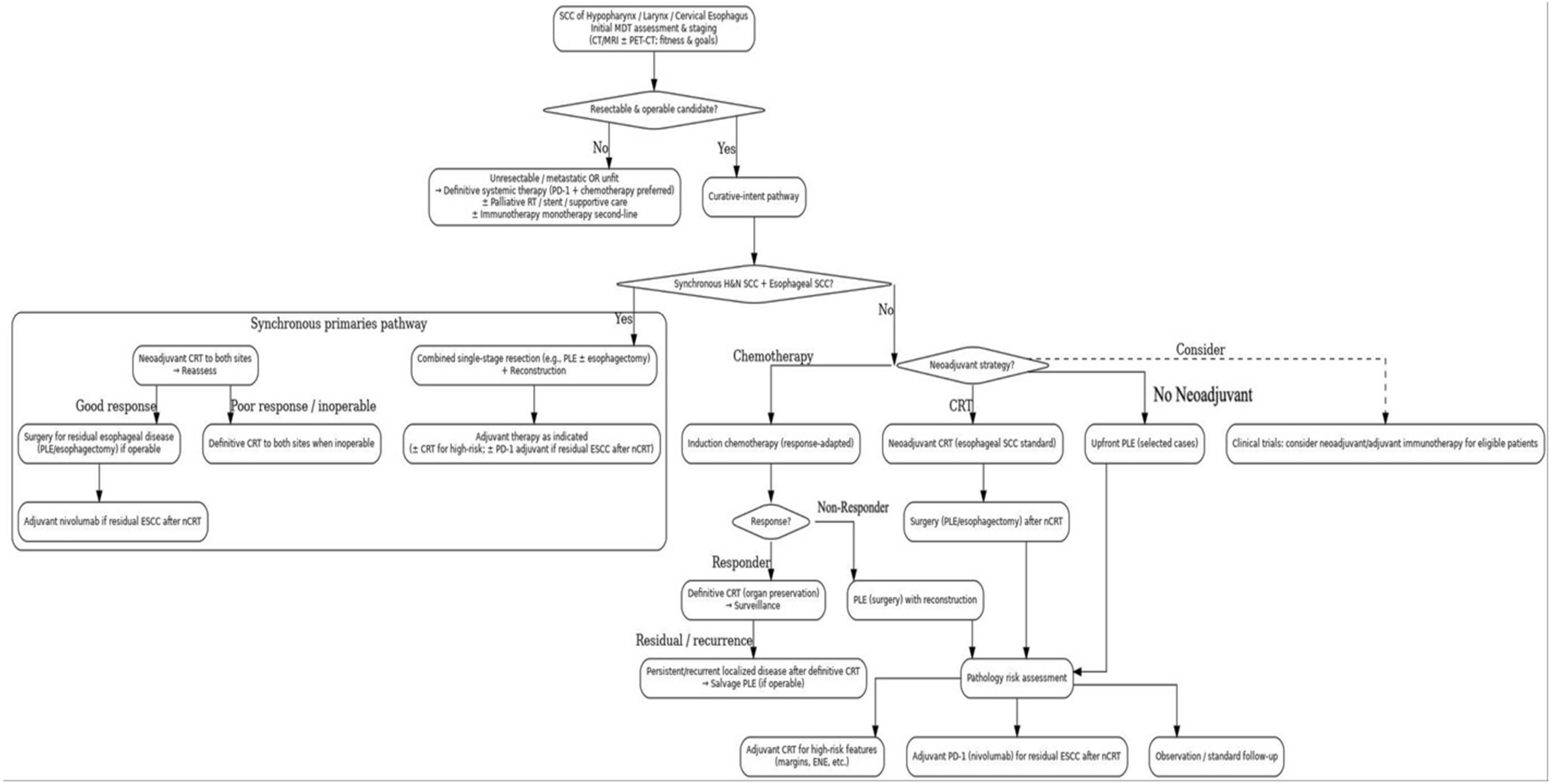

Figure 1. *Multimodal Treatment Algorithm for SCC of the Hypopharynx, Larynx, and Cervical Esophagus [19,20].*

Conclusions

In conclusion, while PLE is a well-established, albeit aggressive, procedure, its role is being continually refined in the context of advancing non-surgical therapies. The future role of PLE will be shaped by advances in patient selection, systemic therapy and surgical innovation. Efforts are underway to refine selection through molecular profiling, imaging, and response-adapted strategies, which may help identify patients most likely to benefit from surgery versus organ-preserving approaches. Immunotherapy is rapidly transforming the treatment landscape, poised to establish new standards of perioperative care. The future likely holds a more nuanced integration of surgery with targeted therapies like immunotherapy, improved safety through minimally invasive techniques and better tissue healing, and hopefully better survival and functional outcomes as a result. Finally, comparative effectiveness research will be critical to define the relative value of surgery versus definitive CRT in specific subsets of patients, particularly as systemic therapies evolve. Through these incremental improvements and innovations, the hope is that we can improve the cure rates of these challenging cancers while minimizing the impact of treatment on patients' lives. These developments underscore a future in which PLE is more personalized, safer, and integrated within multimodal treatment paradigms.

List of Abbreviations

Abbreviation	Full term
ALT	Anterolateral thigh (ALT)
CRT	Chemoradiotherapy (CRT)
DFS	Disease-free survival (DFS)
ESCC	Esophageal squamous cell carcinoma (ESCC)
FU	Fluorouracil (FU)
HNSCC	Head and neck squamous cell carcinoma (HNSCC)

27

Abbreviation	Full term
ICI	Immune checkpoint inhibitor (ICI)
MIPLE	Minimally invasive pharyngolaryngoesophagectomy (MIPLE)
OS	Overall survival (OS)
PD-1	Programmed cell death protein 1 (PD-1)
PD-L1	Programmed death-ligand 1 (PD-L1)
PLE	Pharyngolaryngoesophagectomy (PLE)
QoL	Quality of life (QoL)
SCC	Squamous cell carcinoma (SCC)
TEP	Tracheoesophageal puncture (TEP)
USA	United States of America (USA)

Declarations

Ethics approval and consent to participant: Not applicable.

Consent for publication: Not applicable.

Availability of data and materials: Data sharing is not applicable to this article as no datasets were generated or analysed during the current study. All data discussed in this review are available from the cited published literature.

Competing interests: The authors declare that they have no competing interests.

Funding: This research received no specific grant from any funding agency in the public, commercial, or not-for-profit sectors.

1 Authors' contributions: Major author contributed to the conception and design of the review, performed the literature search, drafted the manuscript and critically revised the manuscript for important intellectual content. All authors read and approved the final manuscript.

Acknowledgements: Not applicable.

Authors' information: Not applicable.

References

1. Jung K, Narwal M, Min SY, Keam B, Kang H: Squamous cell carcinoma of head and neck: what internists should know. Korean J Intern Med 2020;35:1031–1044.
2. Machiels JP, Leemans CR, Golusinski W, Grau C, Licitra L, Gregoire V; EHNS Executive Board; ESMO Guidelines Committee; ESTRO Executive Board: Squamous cell carcinoma of the oral cavity, larynx, oropharynx and hypopharynx: EHNS-ESMO-ESTRO clinical practice guidelines for diagnosis, treatment and follow-up. Ann Oncol 2020;31:1462–1475.
3. Wong I, Law S: The management of mid and proximal oesophageal squamous cell carcinoma. Best Pract Res Clin Gastroenterol 2018;36–37:85–90.
4. Lee Y, Yun J, Jeon YJ, et al: Surgical outcomes of cervical esophageal cancer: a single-center experience. J Chest Surg 2024;57:62–69.
5. Maier A, Pinter H, Tomaselli F, et al: Retrosternal pedicled jejunum interposition: an alternative for reconstruction after total esophagogastrectomy. Eur J Cardiothorac Surg 2002;22:661–665.
6. Butskiy O, Rahmanian R, White RA, et al: Revisiting the gastric pull-up for pharyngoesophageal reconstruction: a systematic review and meta-analysis of mortality and morbidity. J Surg Oncol 2016;114:907–914.
7. Jang JY, Kim EH, Cho J, et al: Comparison of oncological and functional outcomes between initial surgical versus non-surgical treatments for hypopharyngeal cancer. Ann Surg Oncol 2016;23:2054–2061.

8. Sreehariprasad AV, Krishnappa R, Chikaraddi BS, Veerendrakumar K: Gastric pull-up reconstruction after pharyngo-laryngo-esophagectomy for advanced hypopharyngeal cancer. Indian J Surg Oncol 2012;3:4–7.
9. Morita M, Saeki H, Ito S, et al: Technical improvement of total pharyngo-laryngo-esophagectomy for esophageal and head and neck cancer. Ann Surg Oncol 2014;21:1671–1677.
10. Mariette C, Markar SR, Dabakuyo-Yonli TS, et al: Hybrid minimally invasive esophagectomy for esophageal cancer. N Engl J Med 2019;380:152–162.
11. Nuytens F, Dabakuyo-Yonli TS, Meunier B, et al: Five-year survival outcomes of hybrid minimally invasive esophagectomy in esophageal cancer: results of the MIRO randomized clinical trial. JAMA Surg 2021;156:323–332.
12. Obermannová R, Alsina M, Cervantes A, et al: Oesophageal cancer: ESMO clinical practice guideline. Ann Oncol 2022;33:992–1004.
13. Sun F, Li X, Lei D, et al: Surgical management of cervical esophageal carcinoma with larynx preservation and reconstruction. Int J Clin Exp Med 2014;7:2771–2778.
14. Woods JFC, Woods RSR, Lennon P, et al: Outcomes of pharyngo-laryngo-esophagectomy and reconstruction: a longitudinal single-institution analysis. J Plast Reconstr Aesthet Surg 2022;75:1567–1572.
15. Argiris A, Lefebvre JL: Laryngeal preservation strategies in locally advanced laryngeal and hypopharyngeal cancers. Front Oncol 2019;9:419.
16. Keam B, Machiels JP, Kim HR, et al: Pan-Asian adaptation of EHNS-ESMO-ESTRO clinical practice guidelines for head and neck squamous cell carcinoma. ESMO Open 2021;6:100309.
17. Homma A, Nakamaru Y, Hatakeyama H, et al: Early and long-term morbidity after minimally invasive total laryngo-pharyngo-esophagectomy. Eur Arch Otorhinolaryngol 2015;272:3551–3556.
18. Kanie Y, Okamura A, Kanamori J, et al: Postoperative complications following pharyngolaryngectomy with total esophagectomy. Ann Otol Rhinol Laryngol 2023;132:770–776.
19. Booka E, Tsubosa Y, Niihara M, et al: Risk factors for complications after pharyngolaryngectomy with total esophagectomy. Esophagus 2016;13:317–322.
20. Park JW, Lee SW: Clinical outcomes of synchronous head and neck and esophageal cancer. Radiat Oncol J 2015;33:172–178.

21. Bich TA, Vuong NL, Cam Tu NCHTN, et al: Long-term survival after total pharyngolaryngoesophagectomy with gastric pull-up reconstruction. Ann Otol Rhinol Laryngol 2023;132:511–518.
22. Booka E, Takeuchi H, Nishi T, et al: Impact of postoperative complications on survival after esophagectomy. Medicine (Baltimore) 2015;94:e1369.
23. Bourmand R, Olsson SE, Fijany A: Tracheoesophageal puncture and quality of life after total laryngectomy: a systematic review and meta-analysis. Laryngoscope Investig Otolaryngol 2024;9:e70050.
24. Lu YA, Tsao CK, Hsin LJ, et al: Long-term assessment of speech and swallowing after J-flap reconstruction. Clin Exp Otorhinolaryngol 2024;17:346–354.
25. Kuhn MA, Gillespie MB, Ishman SL, et al: Expert consensus statement: management of dysphagia in head and neck cancer patients. Otolaryngol Head Neck Surg 2023;168:571–592.
26. Valmasoni M, Pierobon ES, Zanchettin G, et al: Cervical esophageal cancer treatment strategies. Ann Surg Oncol 2018;25:2747–2755.
27. Riba MB, Donovan KA, Ahmed K, et al: NCCN guidelines insights: distress management, version 2.2023. J Natl Compr Canc Netw 2023;21:450–457.
28. Caudell JJ, Gillison ML, Maghami E, et al: NCCN guidelines insights: head and neck cancers, version 1.2022. J Natl Compr Canc Netw 2022;20:224–234.
29. Yang Y, Feng L, Zhong Q, et al: Induction chemotherapy-based organ preservation versus immediate total laryngectomy in advanced hypopharyngeal cancer. Cancer Med 2023;12:17078–17086.
30. Chang CF, Wang LW, Yang MH, Chu PY: Induction chemotherapy followed by transoral laser microsurgery for advanced hypopharyngeal cancer. J Chin Med Assoc 2024;87:803–808.
31. Panda S, Sakthivel P, Gurusamy KS, et al: Treatment options for resectable hypopharyngeal squamous cell carcinoma. PLoS One 2022;17:e0277460.
32. Lefebvre JL, Andry G, Chevalier D, et al: Laryngeal preservation with induction chemotherapy for hypopharyngeal carcinoma: 10-year results of EORTC trial 24891. Ann Oncol 2012;23:2708–2714.
33. Allum WH, Stenning SP, Bancewicz J, et al: Surgery with or without preoperative chemotherapy in esophageal cancer. J Clin Oncol 2009;27:5062–5067.
34. Harris BN, Biron VL, Donald P, et al: Primary surgery versus chemoradiation for advanced hypopharyngeal squamous cell carcinoma. JAMA Otolaryngol Head Neck Surg 2015;141:636–640.

35. Shapiro J, van Lanschot JJB, Hulshof MCCM, et al; CROSS Study Group: Neoadjuvant chemoradiotherapy plus surgery versus surgery alone for oesophageal cancer. Lancet Oncol 2015;16:1090–1098.
36. Sjoquist KM, Burmeister BH, Smithers BM, et al: Survival after neoadjuvant chemotherapy or chemoradiotherapy for resectable oesophageal carcinoma. Lancet Oncol 2011;12:681–692.
37. Kato K, Machida R, Ito Y, et al: Neoadjuvant doublet chemotherapy, triplet chemotherapy, or chemoradiotherapy for oesophageal cancer (JCOG1109). Lancet 2024;404:55–66.
38. Kitagawa Y, Ishihara R, Ishikawa H, et al: Japanese esophageal cancer practice guidelines 2022. Esophagus 2023;20:343–372.
39. Shah MA, Kennedy EB, Catenacci DV, et al: Treatment of locally advanced esophageal carcinoma: ASCO guideline. J Clin Oncol 2020;38:2677–2694.
40. Horichi Y, Shinomiya H, Kitayama M, et al: Survival outcomes in advanced hypopharyngeal squamous cell carcinoma: Japanese registry data. Head Neck 2025;47:2412–2424.
41. Stafford M, Kaczmar J: Presurgical immunotherapy in head and neck squamous cell carcinoma. Cancers Head Neck 2020;5:4.
42. Masarwy R, Kampel L, Horowitz G, et al: Neoadjuvant PD-1/PD-L1 inhibitors in resectable head and neck cancer. JAMA Otolaryngol Head Neck Surg 2021;147:871–878.
43. Burtness B, Harrington KJ, Greil R, et al: Pembrolizumab versus cetuximab for recurrent or metastatic head and neck squamous cell carcinoma (KEYNOTE-048). Lancet 2019;394:1915–1928.
44. Li R, Ye L, Zhu Y, et al: Induction chemotherapy for laryngeal preservation in hypopharyngeal squamous cell carcinoma. Head Neck 2022;44:2018–2029.
45. Kelly RJ, Ajani JA, Kuzdzal J, et al: Adjuvant nivolumab after resection of esophageal cancer. N Engl J Med 2021;384:1191–1203.
46. Qin J, Xue L, Hao A, et al: Neoadjuvant chemotherapy with or without camrelizumab for resectable esophageal squamous cell carcinoma. Nat Med 2024;30:2549–2557.
47. Qin H, Liu F, Zhang Y, et al: Neoadjuvant immunotherapy versus standard neoadjuvant therapy for esophageal cancer. Front Immunol 2023;14:1108213.
48. Sun JM, Shen L, Shah MA, et al; KEYNOTE-590 Investigators: Pembrolizumab plus chemotherapy for advanced oesophageal cancer. Lancet 2021;398:759–771.

49. Huang TQ, Wang R, Fang JG, et al: Induction chemotherapy for hypopharyngeal carcinoma with cervical oesophageal invasion. World J Surg Oncol 2020;18:95.
50. Zhang B, Zhao H, Wu X, et al: Perioperative outcomes of neoadjuvant camrelizumab in esophageal squamous cell cancer. Front Immunol 2023;14:1066527.
